# Supplementary material for: “I live with pain, it cannot go away”: Lived experiences of childhood and adolescent pulmonary tuberculosis survivors — a qualitative study
Source: PLOS Glob Public Health. 2025 Dec 4;5(12):e0005549. doi: 10.1371/journal.pgph.0005549 (PMC12677498; doi:10.1371/journal.pgph.0005549)
Supplement: S1 Table — (PDF) [file pgph.0005549.s001.pdf]

Workshop agenda

Workshop agenda: children 5 to 10 years

Time	Activity	Details
9:00 am-9:30 am	Arrival	Register
9:30 am-10:00 am	Breakfast	
10:00 am-10:15 am	Round of introductions and how are we feeling	Open as a group by saying<br>- How we are feeling today<br>- One thing that we would like to tell the group about you
10:15 am-10:45 am	First activity – body mapping	Divided into groups<br>Caregivers and their children<br>Draw the outline of their life-size bodies on paper sheets. Then highlight, with colour pens, the parts of their bodies affected since they completed their treatment for TB.
10:45 am-11:00 am	Feedback/debrief	Led by parents/caregivers from each group<br>Describe body mappings to the group
11:00 am-11:30 am	Second activity – drawing/painting	Introduction to drawings/paintings<br>- Make a drawing/painting that you feel represents your child's health now.<br>- Think about if you were to tell someone about your child, what picture would you draw/paint?
11:30 am-11:45 am	Feedback/debrief	Led by a representative from each group<br>Tell us about your picture.<br>- What does each picture mean to you?<br>- What do you think your picture says about your child's health?<br>- If you wanted to add something, but didn't, what would you have added?
11:45 am-12:00 pm	Wrap up	How did you find it?<br>Which part of it did you particularly like?
12:00 pm-12:30 pm	Lunch/departure	

Workshop agenda: adolescents 10 to 19 years

Time	Activity	Details
9:00 am-9:30 am	Arrival	Register
9:30 am-10:00 am	Breakfast	
10:00 am-10:15 am	Round of introductions and how are we feeling	Open as a group, by saying<br>- How we are feeling today<br>- One thing that we would like to tell the group about you
10:15 am-10:45 am	First activity – body mapping activity	**Divided into groups**<br>Draw the outline of their life-size bodies on paper sheets. Then highlight, with colour pens, the parts of their bodies affected<br>since they completed their treatment for TB.
10:45 am-11:00 am	Feedback/debrief	**Led by representative from each group**<br>Describe body mappings to the group
11:00 am-11:30 am	Second activity – games with dice and questions	Divided into groups
11:30 am-11:45 am	Feedback/debrief	**Led by representative from each group**<br>Describe a summary of the group conversations and their interpretations
11:45 am-12:00 pm	Snack break	
12:00 pm-12:30 pm	Third activity – collage/drawing/painting	**Divided into groups**<br>Introduction to collages/drawings/paintings<br>- Make a collage/drawing/painting that you feel represents your health now.<br>- Think about if you were to tell someone about yourself, what picture would you make/draw/paint?
12:30 am-12:45 am	Feedback/debrief	**Led by representative from each group**<br>Tell us about your picture.<br>- What does each picture/visual/words mean to you?<br>- What do you think your picture says about your health?<br>- If you wanted to add something, but didn't, what would you have added?
12:45 am-1:00 pm	Wrap up	How did you find it?<br>Which part of it did you particularly like?
1:00 pm-1:30 pm	Lunch/departure	
