# Supplementary material for: “I live with pain, it cannot go away”: Lived experiences of childhood and adolescent pulmonary tuberculosis survivors — a qualitative study
Source: PLOS Glob Public Health. 2025 Dec 4;5(12):e0005549. doi: 10.1371/journal.pgph.0005549 (PMC12677498; doi:10.1371/journal.pgph.0005549)
Supplement: S1 Text — (PDF) [file pgph.0005549.s003.pdf]

Interview Topic Guides

Interview Topic Guide (adolescent)

Date	
Time	
Duration	
Location	
Participant ID	
Audio file ID	
Name of interviewer	
Name of interpreter	

Purpose: The goal of this guide is to help facilitate one-on-one interviews with children and adolescents involved in the Childhood TB Sequel study. The topic guide is aimed at exploring the lived experiences of children and adolescents after TB. This guide will be used flexibly during the discussion.

Preamble **(to be read by researcher at the start of the interview)**: Today is the (insert date [day xxth Xxx xxxx]) and it is (insert time XX:XX). This is an interview with a child/adolescent participant who is a part of the Childhood TB Sequel Study. Thank you for your time and letting me talk to you today. Today I would like to talk to you ___________ (insert participant's name) about your experiences since completing treatment for TB. Before we get started, I want you to know that everything we talk about today will remain anonymous. We may write a publication or report. However, all the quotes will be anonymised to protect your identity. These reports and/or publications will not include any names or other personal information. Remember that there are no right or wrong answers and I am sincerely interested in your personal experiences in order for us to better understand how TB may affect your daily lives. May I also remind you that we are audio recording this discussion and ask that you speak loud and clear. Do you have any questions before we start?

Stage 1: Background information about the participant and family *(Note: The researcher can provide the participant with a colouring-in sheet or blank sheet of paper to draw while interviewing)*

Now we would like to ask you ________ (insert the participant's name) some questions. You can write or draw your responses. Does that sound good to you?

- Please briefly tell me about yourself *(Probes: Name, age, grade, likes, dislikes, dreams)*
- Tell me about the things that make you happy. (Probes: friends, family, playing, school etc.)
- Tell me about the things that make you sad.
- Who do you live with? *(Probes: who lives with you? Mother, father, brothers, sisters, grandmother, grandfather, aunties, uncles, cousins, etc. Does the other family live close by? Who takes care of you?*
- What have your experiences been since you started coming to MRC *(Probes: How long have you been part of the study? What makes it easy to come back? What makes it difficult to come back? What procedures do you appreciate (e.g. because it seems important), and which procedures do you fear?)*

- How did you know you have TB *(Probes: how did you feel? What was going on in your mind? Where did you feel pain?)*
- Who was the first person who told you that you were ill? *(Probes: Mother, Father, Grandmother, Grandfather, Sister, Brother).*
- How have you felt since you completed your treatments?

Stage 2: Lived experiences

Now, we would like to know more about your experiences since you completed your TB treatment.

- Are you going to school?
- Tell me about your school. *(Probes: What do you like about school? What don't you like about school?)*
- Are you involved in any school activities? Tell me about the activities you do in school. *(Probes: Can you run and play like other friends? Has this changed after the disease?)*
- Tell me about your teachers.
- Do you have friends at school/ community? Please tell me about your friends at school/ community.
- If you think about your siblings and friends, tell me about how they reacted when they heard you were ill. *(Probes: How did they act towards you? How were you treated compared to others? How did that make you feel? What were you not allowed to do?)*
- Please take me through a day in your life. *(Probes: from when you wake up till you go to bed. Time you wake-up, breakfast, school/day-care, activities – playing, TV, sport etc.)*
- Tell me about the days that you are feeling well. *(Probes: How does that make you feel? What do you like doing? Games you like to play)*
- Tell me about the days that you are not feeling well. *(Probes: How does that make you feel? What are you not allowed to do? are your usual activities such as sports, playing, running and/or riding a bike restricted?)*
- Please tell me about what your life was like when you were ill. *(Probes: How did that make you feel? What were you not allowed to do? Were your usual activities, such as sports, playing, running and/or riding a bike, restricted? What games did you like to play but could not because you were ill? How did it affect your concentration in class? How did it affect your school attendance? How much of school did you miss? Did you have to drop out of school?)*
- Now, please tell me about what your life is like after TB. *(Probes: How does it make you feel? What do you like doing now that you couldn't do when you were ill? How well do you get along with other children in school or at home? Are you able to go back to school? Did you have to repeat a class? How well are you performing in school?)*
- How do your siblings and friends think about you now after TB? *(Probes: How do they act towards you? How are you treated compared to others? How does that make you feel? What are you not allowed to do?)*
- Please tell me anything else you would like me to know about how TB has changed your life.

Thank you for your time.

Provide transportation refunds and refreshments.

Interview Topic Guide (caregiver)

Date	
Time	
Duration	
Location	
Participant ID	
Audio file ID	
Name of interviewer	
Name of interpreter	

Purpose: The goal of this guide is to help facilitate one-on-one interviews with caregivers of children involved in the Childhood TB Sequel study. The topic guide is aimed at exploring the lived experiences of children and adolescents after TB. This guide will be used flexibly during the discussion.

Preamble **(to be read by researcher at the start of the interview):** Today is the (insert date [day xxth Xxx xxxx]) and it is (insert time XX:XX). This is an interview with a caregiver of child/adolescent participant who is a part of the Childhood TB Sequel Study. Thank you for your time and letting me talk to you today. Today, I would like to talk to you ____________ (insert caregiver's name) about your child's ____________ (insert participant's name) experiences since completing treatment for TB. Before we get started, I want you to know that everything we talk about today will remain anonymous. We may write a publication or report. However, all the quotes will be anonymised to protect your identity. These reports and/or publications will not include any names or other personal information. Remember that there are no right or wrong answers and I am sincerely interested in your personal experiences in order for us to better understand how TB may affect your child's daily life. May I also remind you that we are audio recording this discussion and ask that you speak loud and clear. Do you have any questions before we start?

Stage 1: Background information about the participant and family

Now we would like to ask you ________ (insert the caregiver's name) some questions.

- Please briefly tell me about yourself *(Probes: Name, age, grade, likes, dislikes, dreams)*
- Please briefly tell me about your household. *(Probes: who lives with you? Mother, father, brothers, sisters, grandmother, grandfather, aunties, uncles, cousins, etc. Does the other family live close by? Who takes care of the child in the study?*
- Please tell me about your experience since you started coming to MRC. *(Probes: How long has your child been part of the study? What makes it easy to come back? What makes it difficult to come back? What procedures do you appreciate (e.g. because it seems important), and which procedures do you fear?)*
- Please tell me about the first time your child felt sick with TB *(Probes: Where did it hurt? Symptoms?)*
- Please tell me who was the first person to tell you that your child is sick. *(Probes: Mother, Father, Grandmother, Grandfather, Sister, Brother).*
- Please tell me about any concerns that you may have about your child's life after TB.

Stage 2: Lived experiences

Now, we would like to know more about your child's experiences since they completed their TB treatment.

- Tell me about your child's school. *(Probes: What do they like about school?, What don't they like about school?)*
- Tell me about their teachers.
- Please tell me about their friends in school and at home.
- Please take us through a typical day in the life of the child. *(Probes: from the moment the child wakes up till bedtime for the child. What time does the child wake up? Breakfast-together? School/ daycare? Dinner? Activities? TV?)*
- Please tell me about what their life was like when they were ill. *(Probes: How did that make you feel? What were they not allowed to do? Were their usual activities, such as sports, playing, running and/or riding a bike, restricted? What games did they like to play but could not because they were ill? How did it affect their school performance? How did it affect their school attendance? How much of school did they miss? Did they have to drop out of school?)*
- If you think about their siblings and friends, tell me about how they reacted when they heard your child was ill. *(Probes: How did they act towards your child? How was your child treated compared to others? How did that make you feel? What was your child not allowed to do?)*
- Now, please tell me about what your child's life is like after TB. *(Probes: What are your fears? What do they like doing now that they couldn't do when they were ill? How well do they get along with other children in school or at home? Are they able to go back to school? Did they have to repeat a class? How well are they performing in school?)*
- How do their siblings and friends think about them now after TB? *(Probes: How do they act towards your child? How are they treated compared to others? How does that make you feel? What are they not allowed to do?)*
- Tell me about the days that your child has been feeling well. *(Probes: How does that make you feel? What do they like doing? Games they like to play)*
- Tell me about the days that your child is not feeling well now. *(Probes: How does that make you feel? What are they not allowed to do? are their usual activities such as sports, playing, running and/or riding a bike restricted?)*
- Please tell me anything else you would like me to know about how TB has changed your child's life.

Thank you for your time.

Provide transportation refunds and refreshments.
