# Supplementary material for: “I live with pain, it cannot go away”: Lived experiences of childhood and adolescent pulmonary tuberculosis survivors — a qualitative study
Source: PLOS Glob Public Health. 2025 Dec 4;5(12):e0005549. doi: 10.1371/journal.pgph.0005549 (PMC12677498; doi:10.1371/journal.pgph.0005549)
Supplement: Attachment — Submitted filename: Response to reviewers.docx [file pgph.0005549.s007.docx]

# Reviewer: 1

| **Comments to the Author** | **Response to reviewer** |
| --- | --- |
| Consider to rephrase the title for more clarity: “I live with pain, it cannot go away”: Lived experiences of childhood and adolescent pulmonary tuberculosis survivors — a qualitative study | We have rephrased the title in line with the reviewer’s suggestion. It now reads as follows:  “I live with pain, it cannot go away”: lived experiences of childhood and adolescent pulmonary tuberculosis survivors — a qualitative study.” |
| **Introduction** |  |
| Consider constructing the theoretical framework or perspective guiding the qualitative inquiry (e.g., illness narrative, trauma theory, etc.). | We have clarified in the Introduction (**lines 72-75**) the theoretical perspective guiding our qualitative inquiry, explaining that we employ phenomenology within an illness-narrative framework focused on meaning-making, identity, and recovery after illness. |
| Clarify how this study builds upon or diverges from existing qualitative TB studies | We have clarified in the Introduction (**lines 54-64**) how our study extends existing qualitative TB research. Unlike previous studies, which mainly examine adults and their treatment experiences, our work focuses on children and adolescents during the post-treatment period, emphasizing their developmental and psychosocial adaptations after TB cure. |
| **Methods** |  |
| Provide more information on the demographic characteristics of participants (age ranges, gender distribution, time since treatment completion, etc). | We have added a paragraph to the Methods section (**lines 120-126**) that provides additional demographic details, including age range, gender distribution, and time since treatment completion. Additionally, we inserted a linking sentence in the Results – Characteristics of the Sample section (**lines 265-269**) to maintain consistency and provide context regarding the timing of participants’ reflections. |
| Describe researcher positionality to enhance trustworthiness. | We have revised the 'Researchers’ Perspectives and Reflexivity' section (**lines 226-236**) to explicitly describe the research team’s positionality. The updated text clarifies the complementary roles of the lead researchers—EN as a clinician–researcher and ON as a social scientist—and explains how their engagement with the study context, professional identities, and reflexive practices contributed to the trustworthiness of the analysis. |
| Clarify if any software (e.g., NVivo, Atlas.ti) was used for coding. | We have clarified that NVivo software was used to support coding and data management (**lines 212-213**)**.** |
| **Results** |  |
| Ensure each theme is analytically developed, not just descriptive. | We have strengthened the Results section by adding interpretive linking sentences, brief within-theme syntheses, and examples of contrasting cases to deepen analytical development and clarify the mechanisms underlying each theme. (**lines 281-284; 302-309; 343-348; 350-353; 407-409; 422-423; and 434-436**). |
| Discuss any deviant or contradictory cases if applicable. | We have included brief statements in the Results section to highlight deviant or contrasting cases, such as participants who felt fully recovered and those who saw their illness as motivation to pursue medical careers, thereby capturing the diversity of post-TB experiences and responses. (**lines 343-348; 458-460**). |
| **Discussion** |  |
| Elaborate on how findings may inform rehabilitation frameworks or public health interventions. | We have revised the Discussion section to include recommendations for low-cost interventions with insights on how our findings can inform rehabilitation and public health frameworks. The new paragraph (**lines 529-536**) presents integrated and feasible approaches such as school reintegration, community engagement, and psychosocial follow-up within a practical, rehabilitation-focused model. |
| Include comparative findings from other global regions. | Thank you for your suggestion. We designed our study to explore in-depth and context-specific paediatric post-TB experiences in The Gambia. Although we reviewed the available literature to frame our findings, there is a notable lack of qualitative research worldwide on the lived experiences of children and adolescents after TB treatment. Therefore, comprehensive comparisons across regions are not yet feasible. Our study contributes to filling this gap by providing foundational evidence from a high-burden African context. |
| **Conclusion:** Summarize actionable recommendations for clinicians, educators, and policymakers. | We have included a clear, concise summary of actionable recommendations for clinicians, educators, and policymakers in the Conclusion (**lines 562-565**). |
| **References:** Check all references to comply with journals referencing guidelines | We have done this. |

# Reviewer: 2

| **Comments to the Author** | **Response to reviewer** |
| --- | --- |
| At the outset, I must congratulate the authors for the well-structured and conducted study about such an under-researched topic like the lived experiences of children and adolescents with TB. The work’s focus on persistent sequelae, psychosocial implications, and post-TB aspirations among youth is relevant and well aligned with global health priorities. Study which has a child protection specialist during the IDI showed the authors inclination towards upholding principles of Child Rights and protection and must be definitely lauded as a great feat! These are few of my concerns and comments. | We thank the reviewer for this comment. We now provide a point-by-point response to the reviewer’s comments below. |
| **General comments:** |  |
| 1. Appreciate the detailed process of the study performed and the age-appropriate interventions and assessment tools used to understand the underlying concerns in children and adolescents. The use of IPA as methodology with participatory workshops was commendable. | We thank the reviewer for their positive feedback and recognition of our methodological approach. |
| 2. Would recommend a simple clarification on how these participants were chosen so as to understand if any eligible participants declined and it was probably linked to poor experiences during TB care. | We have clarified the participant selection process and addressed whether any eligible individuals declined participation. We included a statement noting that, while a few participants were unavailable due to relocation or scheduling conflicts, none refused participation due to negative TB care experiences (**lines 120-126**). |
| 3. Would recommend a more detailed explanation of the various patterns seen in the pictographic representations of physical ailments seen. | We have expanded the description accompanying Figure 2 to include a brief interpretation of the visual patterns observed in the body-mapping exercise, explaining how participants used colour, shading, and repetition to represent areas of pain and fatigue. |
| 4. A general description or subgroup analysis to look at PTB Vs EPTB Vs Disseminated TB in the experiences would be useful to look at models to improve outcomes tailored by the type of TB encountered. | Thank you for your thoughtful suggestion. This study was limited to survivors of pulmonary tuberculosis (PTB) because it was embedded in the Childhood TB Sequel cohort, which recruited only children and adolescents treated for drug-sensitive pulmonary TB. As a result, cases of extrapulmonary or disseminated TB were not part of our analysis. We agree that comparative qualitative studies exploring the lives of survivors with other TB forms (e.g., EPTB and disseminated TB) would be valuable for developing tailored post-TB care models and represent an important direction for future research. |
| 5. Would be advisable to clarify and tabulate participant and caregiver quotations separately as it becomes difficult to understand age-based responses and perspectives. | Thank you for this thoughtful suggestion. We agree that distinguishing between the voices of participants and caregivers is important for interpretation. To address this, we have added a clarifying statement in the Results section (**lines 270-272**), noting that all quotations are now identified by participant type and age (e.g., “17-year-old male,” “caregiver of an 11-year-old female”).  We have not organised quotations in a separate table because this study uses an interpretative phenomenological approach, wherein quotations are intentionally embedded within thematic narratives. This integration preserves the depth, flow, and contextual richness of participants’ lived experiences. We believe this approach maintains analytic integrity and clarity while effectively capturing age- and role-specific perspectives. |
| 6. Would recommend a small note on discussion of the various low-cost high impact interventions you would suggest based on your findings to strengthen TB care in Gambia. | We have expanded the Discussion section to include a paragraph outlining feasible, low-cost, and high-impact interventions to strengthen TB care in The Gambia (**lines 529-536**). These additions, comprising school reintegration, community-led stigma reduction, and psychosocial counselling, are directly informed by our study findings and align with the paper’s emphasis on holistic, age-appropriate, and gender-responsive post-TB support. |
| 7. At most reflections, it is difficult to understand if the reflections are during the time of disease or treatment or now in Post TB life since some of the experiences are clouded by the concerns while on treatment (drugs related, disease pathology related) which are rarely of any concerns post treatment. It may be imperative to speak about the lived experience as not just the post care period but also during the diagnosis, treatment and follow up period. | We agree that clarifying the temporal context of participants’ reflections enhances the analytical depth. To address this, we added framing sentences in the Results section to explicitly state that participants’ accounts often combine memories from their diagnosis and treatment periods with their current post-TB experiences. These revisions clarify that the lived experience is seen as a continuum encompassing illness, treatment, and life after treatment (**lines 265-269**). |
| Looking forward to your edited manuscript and hopeful that the outcome will be definitely a good read! | We sincerely thank the reviewer for their encouraging feedback and insightful comments. Their careful review and suggestions have significantly helped us strengthen the manuscript by improving its clarity and analytical depth. |
